# Thiamine Deficiency After Bariatric Surgery: Early Neurological Complications and Nutritional Monitoring

**DOI:** 10.7759/cureus.100637

**Published:** 2026-01-02

**Authors:** Sara Remelhe Sá, João Lagarteira, Rita Pera, Ana Figueiredo, Cristiana Batouxas

**Affiliations:** 1 Internal Medicine Department, Unidade Local de Saúde do Nordeste, Bragança, PRT

**Keywords:** bariatric surgery, nutritional deficiency, postoperative complications, sixth cranial nerve palsy, thiamine deficiency

## Abstract

Thiamine (vitamin B1) deficiency is a recognized complication after bariatric surgery (BS) that can lead to severe neurological manifestations. Early identification is critical to prevent irreversible complications. We report a 23‑year‑old woman who presented two months after BS-Roux-en-Y gastric bypass (RYGB) with diplopia and limitation of ocular abduction in the right eye, consistent with isolated sixth cranial nerve palsy. She had no history of head trauma, infection, or prior neurological disease. Cranial CT angiography and brain MRI excluded ischemic, hemorrhagic, vascular, or demyelinating lesions. Laboratory tests revealed thiamine deficiency (20 ng/mL) and folic acid deficiency (1.7 ng/mL). The patient was treated with thiamine 600 mg/day, folic acid 5 mg/day, and cyanocobalamin 1 mg/day, resulting in complete resolution of symptoms.

## Introduction

Obesity is a chronic, multifactorial disease with a high global prevalence, and it is associated with a significant increase in morbidity and mortality [1]. Its incidence has been rising markedly over recent decades, currently representing a major public health challenge [1,2]. Despite the various therapeutic approaches available, including lifestyle modification and pharmacological treatment, sustained weight loss remains difficult to achieve and maintain for a large proportion of patients [1,3]. In this context, bariatric surgery (BS) has established itself as an effective therapeutic intervention for weight reduction in individuals with severe obesity, also contributing to the improvement of various metabolic comorbidities [1-3]. However, despite its effectiveness and relative safety, BS can be associated with complications, among which neurological complications stand out, which can involve both the central nervous system (CNS) and the peripheral nervous system (PNS) [2,4]. Among the main causes of these neurological complications is micronutrient deficiency, with particular emphasis on vitamin B1 (thiamine) [1-5].

Thiamine deficiency is a potentially serious and frequently underdiagnosed condition that may occur early after surgery, with clinical manifestations reported as early as the 15th postoperative week [2,6]. The prevalence of thiamine deficiency after BS has been reported to be substantial, with pooled estimates showing around 9-19% in the early postoperative months [1,3]. Thiamine deficiency results from decreased intake, malabsorption, and persistent vomiting, and can deplete body stores within weeks due to low tissue reserves and high metabolic demand [2,6-8]. This nutritional deficiency can result in severe neurological conditions, such as Wernicke’s encephalopathy, peripheral neuropathy, and other irreversible neurological changes, if not identified and treated promptly [4-8].

Given the severity of potential complications associated with thiamine deficiency, its prevention is essential through appropriate nutritional monitoring and supplementation in the postoperative period. Clinical practice guidelines for BS recommend routine surveillance of micronutrient levels, including thiamine, folate, and vitamin B12, at regular intervals in the first year after surgery, and tailored supplementation as needed [9,10]. We report a case of isolated sixth cranial nerve palsy secondary to thiamine deficiency following BS, highlighting the importance of early recognition and treatment.

## Case presentation

A 23-year-old woman with a history of obesity underwent BS-Roux-en-Y gastric bypass (RYGB) in September 2023. Since the procedure, she had been taking oral multivitamin supplements containing iron, calcium, zinc, folic acid, vitamin D, and vitamin B12. After 2 months, she presented to the emergency department with a five-day history of diplopia and dizziness. She denied any history of head trauma, recent infections, or exposure to infectious contacts. On physical examination, she exhibited diplopia, worsened when viewing distant objects, and limitation of ocular abduction in the right eye (RE), consistent with isolated right sixth cranial nerve palsy. No other neurological deficits were identified.

**Figure 1:**
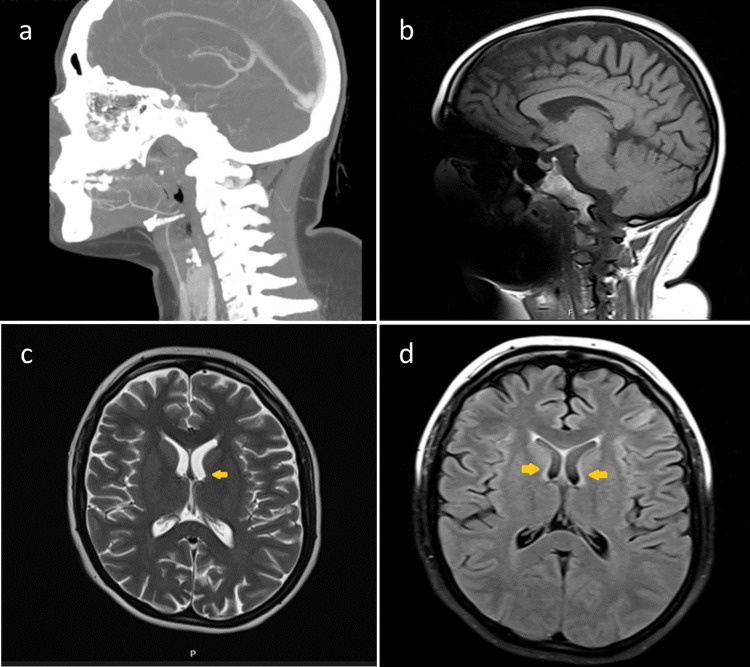
Neuroimaging of a patient with thiamine deficiency. Sagittal CT (a) showing no significant bony abnormalities; sagittal T1-weighted MRI (b) with preserved brain anatomy; axial T2-weighted MRI (c) showing symmetric hyperintensities in the dorsomedial thalami; axial FLAIR MRI (d) demonstrating hyperintensities in the dorsomedial thalami and periaqueductal region, consistent with thiamine deficiency.

A cranial CT angiography was performed, excluding ischemic or hemorrhagic events, vascular abnormalities, and mass-effect lesions. Brain magnetic resonance imaging (MRI) did not reveal findings suggestive of multiple sclerosis (Figure 1).

Laboratory evaluation revealed a hemoglobin level of 14 g/dL, with no leukocytosis and no significant elevation of inflammatory markers (erythrocyte sedimentation rate 35 mm/h; C-reactive protein 2.25 mg/dL) (Table 1).

**Table 1:** Laboratory test results. ESR: erythrocyte sedimentation rate; CRP: c-reactive protein.

Parameter	Result	Normal Range
Hemoglobin (g/dL)	14	12.0-16.0
Platelets (x10^9^/L)	283	150-450
ESR (mm/h)	35	0-20
Leukocytes (x10^9^/L)	7.24	4.0-11.0
Neutrophils (x10^9^/L)	4.52	2.0-7.0
CRP (mg/dL)	2.25	<3
Folic acid (ng/mL)	1.7	3.0-20.5
Vitamin B1 (ng/mL)	20	28.0-85.0

Lumbar puncture revealed cerebrospinal fluid with a normal macroscopic appearance. Analyses, including meningitis/encephalitis PCR, immunofixation, and oligoclonal band testing, were negative, thereby excluding infectious or autoimmune inflammatory etiologies. The ophthalmological examination confirmed diplopia, without a visual acuity deficit. The fundus examination was normal. The Hess-Lancaster test revealed isolated paresis of the right VI cranial nerve, and ocular occlusion was instituted with a plan for re-evaluation after three months.

After ruling out major structural, inflammatory, and infectious causes and considering the context of recent BS, an extensive analytical study was requested. This revealed severe folic acid deficiency (1.7 ng/mL) and vitamin B1 deficiency (thiamine: 20 ng/mL) (Table 1).

Treatment was initiated with folic acid 5 mg/day, cyanocobalamin 1 mg/day intramuscularly for five days, followed by monthly maintenance, and thiamine 200 mg intravenously every eight hours for five days. The patient exhibited progressive neurological improvement, with partial recovery by day four and complete resolution of deficits by day 10 of supplementation. At the time of discharge, she had complete resolution of symptoms. In a follow-up consultation with Internal Medicine and Neurology one month after discharge, she was asymptomatic, without neurological deficits, and with normalized vitamin levels.

## Discussion

Thiamine (vitamin B1) deficiency is a known, though often underdiagnosed, complication following BS, particularly in techniques with a malabsorptive component, but it can also occur in predominantly restrictive procedures due to low intake, persistent vomiting, or poor adherence to vitamin supplementation [1,2,5,8]. In this clinical case, a young woman developed paresis of the VI cranial nerve (with diplopia and limitation of ocular abduction) about two months after BS. The absence of classic signs of Wernicke’s encephalopathy, namely mental confusion and ataxia, can make the diagnosis more challenging, as focal neurological manifestations, such as isolated cranial nerve palsies, have been described in the literature as atypical but possible presentations of thiamine deficiency [6,7].

Although the patient received multivitamin supplementation postoperatively, marked deficiencies in thiamine and folic acid were observed. This highlights that generic supplementation may be insufficient, particularly when not tailored to the type of surgery and the patient’s clinical course [3,9,10]. Folic acid deficiency, although less frequently associated with isolated neurological manifestations, may act synergistically and worsen the clinical presentation [3].

The favourable evolution following the initiation of treatment with thiamine, folic acid, and cyanocobalamin reinforces the importance of early intervention. The literature emphasizes that thiamine replacement should be initiated immediately whenever there is clinical suspicion, as delays in treatment can result in permanent neurological sequelae [6,8].

Finally, this case underscores the need for strict nutritional surveillance in the follow-up of patients undergoing BS. Major international guidelines recommend periodic monitoring of thiamine, folate, vitamin B12, and other micronutrients during the first 6-12 postoperative months, or whenever symptoms suggestive of nutritional deficiencies arise [9,10].

## Conclusions

This clinical case demonstrates that severe nutritional deficiencies may occur in the postoperative period following BS despite multivitamin supplementation and may present with atypical focal neurological manifestations. Thiamine deficiency should therefore be considered in the differential diagnosis of any bariatric patient presenting with neurological symptoms, even in the absence of the classic Wernicke encephalopathy triad. Early recognition and prompt initiation of targeted supplementation are crucial to prevent irreversible complications. Furthermore, this case underscores the importance of rigorous multidisciplinary follow-up after BS, including individualized supplementation strategies, patient education, and regular laboratory monitoring.
